# Character Strengths Profiles in Medical Professionals and Their Impact on Well-Being

**DOI:** 10.3389/fpsyg.2020.566728

**Published:** 2020-12-23

**Authors:** Alexandra Huber, Cornelia Strecker, Timo Kachel, Thomas Höge, Stefan Höfer

**Affiliations:** ^1^Department of Medical Psychology, Medical University of Innsbruck, Innsbruck, Austria; ^2^Institute of Psychology, University of Innsbruck, Innsbruck, Austria; ^3^Department of Psychiatry, Psychotherapy and Psychosomatics, University Hospital of Psychiatry II, Medical University of Innsbruck, Innsbruck, Austria

**Keywords:** character strengths profiles, VIA-classification, medical students, physicians, well-being, work engagement

## Abstract

Character strengths profiles in the specific setting of medical professionals are widely unchartered territory. This paper focused on an overview of character strengths profiles of medical professionals (medical students and physicians) based on literature research and available empirical data illustrating their impact on well-being and work engagement. A literature research was conducted and the majority of peer-reviewed considered articles dealt with theoretical or conceptually driven ‘virtues’ associated with medical specialties or questions of ethics in patient care (e.g., professionalism, or what makes a good physician). The virtues of compassion, courage, altruism, and benevolence were described most often. Only a limited number of papers addressed character strengths of medical students or physicians according to the VIA-classification. Those articles showed that the VIA-character strengths *fairness, honesty*, *kindness*, and *teamwork* were considered most often by respondents to be particularly important for the medical profession. Available cross-sectional (time span: six years) and longitudinal (time span: three years) data regarding VIA-character strengths profiles of medical professionals were analyzed (*N* = 584 medical students, 274 physicians). These profiles were quite homogenous among both groups. The character strengths *fairness*, *honesty*, *judgment*, *kindness*, and *love* had the highest means in both samples. Noteworthy differences appeared when comparing medical specialties, in particular concerning general surgeons and psychiatrists, with the former reporting clearly higher levels of e.g., *honesty* (*d* = 1.02) or *prudence* (*d* = 1.19). Long-term results revealed significant positive effects of character strengths on well-being and work engagement (e.g., *perseverance* on physicians’ work engagement) but also significant negative effects (e.g., *appreciation of beauty and excellence* on students’ well-being). Further, *hope* was significantly associated both positively with physicians’ well-being and negatively with students’ work engagement, possibly indicating specific issues concerning medical education or hospital working conditions. According to the modern-day physician’s pledge, medical professionals should pay attention to their own well-being and health. Therefore, promoting self-awareness and character building among medical professionals could be a beneficial strategy.

## Introduction

Character strengths are inherent in all humans. They are reflected in everyday thoughts, attitudes, and behaviors, and have a positive relation to one’s well-being (Peterson and Seligman, 2004). The discipline of Positive Psychology (Seligman and Csikszentmihalyi, 2000) defines character strengths as a group of positively valued and moral traits that an individual can possess, enabling growth, flourishing and moral excellence (Seligman, 2002). The ‘Values in Action’ (VIA) classification describes 24 character strengths, assigned to six virtues (courage, humanity, justice, temperance, transcendence, wisdom) that have been theoretically considered as being important for over 3000 years across different religions, cultures, and traditions (Peterson and Seligman, 2004). Seligman (2002) suggests that individuals ought to utilize their character strengths by enforcing them according to their life circumstances to obtain well-being and to increase positive benefits. Some character strengths have been identified to be more strongly related with life satisfaction and occupational well-being than others, the so-called ‘happiness strengths’ *curiosity*, *gratitude*, *hope*, *love*, and *zest* (e.g., Peterson and Park, 2006; Buschor et al., 2013; Littman-Ovadia et al., 2016). In another study, *perseverance* and *social intelligence* were most strongly associated with life satisfaction (beside *hope*, *love*, and *zest*) and *humor* playing an important role for well-being (Martínez-Martí and Ruch, 2014). Applying e.g., *gratitude* (Emmons and McCullough, 2003; Machado et al., 2019) or *kindness* (Otake et al., 2006) led to higher levels of well-being, and generally the application of character strengths at work was related to various positive experiences (e.g., pleasure, work engagement, meaning) and job satisfaction (Littman-Ovadia and Steger, 2010; Seligman, 2011; Harzer and Ruch, 2012, 2013). Others identified *appreciation of beauty and excellence, creativity, judgment, love of learning*, and *humility* to be least related with life satisfaction (Park et al., 2004).

Furthermore, specific character strengths might be more prevalent among certain groups of people or professions than among others. Such ‘profiles’ might exist because (1) a certain job rather attracts people with a certain distinct set of character strengths, and (2) shared environments (e.g., study or working conditions, occupational and organizational structures, processes and cultures, trainers/colleagues as role models, etc.) shape individual character strengths in a similar vein toward a ‘collective profile’ (Peterson and Seligman, 2004). Particularly medical students and hospital physicians need to master comprehensive demands and strains. Additionally, they often report impaired well-being or even mental illness raising the question on possible underlying character strengths profiles. Medical students reported more depressive symptoms and higher levels of distress with regard to their health compared to the general United States population (Dyrbye et al., 2014), impaired mental health (e.g., Brazeau et al., 2014) and well-being (e.g., Dunn et al., 2008), or early onset of burnout symptoms (Kachel et al., 2020a). Moreover, origins of recurrent physician burnout were identified with studies showing a prevalence of 45% up to 70% to have these symptoms during medical education at least once (Dyrbye et al., 2008; Ishak et al., 2013) entailing health impairing consequences (e.g., Jackson et al., 2016). Physicians are further confronted with various work demands and job strains (e.g., workload, time pressure, emotional labor, social stressors, cognitive demands; Angerer and Weigl, 2015) and when they feel unwell, the performance of health-care systems as well as patient care can be impaired (e.g., Wallace et al., 2009; Klein et al., 2010). Compared to the general population, an increased burnout risk was reported (resident physicians: 60%; physicians: 51%; Dyrbye et al., 2014), and in addition depression, substance abuse and suicide occurred above-average (Gold et al., 2013). Indeed, medical students and hospital physicians are exposed to challenging circumstances but just therefore, actively pleading for individual positive experiences in terms of applying one’s character strengths could be particularly beneficial for their well-being and health (e.g., Hershberger, 2005).

Therefore, this study aimed to determine possible character strengths profiles of medical professionals based on a focused literature research on medical students’ and physicians’ virtues and VIA-character strengths and own empirical data. Possible differences regarding character strengths profiles of various sub-groups (e.g., age, sex, different medical specialties) will be discussed as well as their respective relevance and relation to medical professionals’ well-being and work engagement.

## Virtues and Character Strengths in the Literature

The science of psychology as it has been practiced until the 1980s/90s needed to be enriched by focusing more on positive aspects of human experiences and behavior (Peterson and Seligman, 2004) as the historically developed imbalance toward studying psychopathology and negative aspects within this discipline (e.g., mental disorders, diagnoses, and treatment; Cassell, 2002; Sheldon and Lyubomirsky, 2004; Harzer and Ruch, 2013) threatened to turn unilateral. Thus, a paradigm shift was heralded by Positive Psychology in the late 1990s by Martin E. P. Seligman as one of its founders. Based on the fundamental virtues of courage, humanity, justice, temperance, transcendence, and wisdom, character strengths were emphasized again (Peterson and Seligman, 2004). These strengths are considered quite stable characteristics of an individual, therefore a theoretical overlap with personality traits is possible (Park et al., 2004; Peterson and Seligman, 2004). However, one conceptual difference between character strengths and personality traits is their normative vs. descriptive perspective on individual differences. Character strengths are positively valued (normative) whereas personality traits are usually described in an unbiased way on continuums (e.g., Five-Factor Model = Big Five; McCrae and Costa, 1997). Only pathological personality aspects were always clearly negatively valued (e.g., Dimensional Assessment of Personality Pathology; Livesley, 2006). The positive, moral valuation of ‘character’ led to its exclusion from personality psychology in the 1930s as Gordon Allport defined that ‘Character is personality evaluated, and personality is character devaluated. Since character is an unnecessary concept for psychology, the term will not appear again in this volume…’ (c.f. Linley et al., 2007). Today’s research is questioning this exclusion as there is new evidence indicating that virtues, and therefore character strengths respectively, are an expression of personality rather than ‘moral reasoning and cognitive development’ (see Cawley et al., 2000). For example, people scoring high on the Big Five dimension ‘agreeableness’ (= being friendly and compassionate vs. challenging/callous) also reported higher levels of *forgiveness, gratitude*, *hope, kindness, prudence* or *self-regulation* (Haslam et al., 2004; Brose et al., 2005; Wood et al., 2008), while ‘neuroticism’ (= being sensitive and nervous vs. resilient/confident) negatively predicted *bravery* and *hope* (Macdonald et al., 2008). The latter study tested a theoretically derived model relating the six VIA-virtues to the Big Five revealing no correlate for the VIA-virtue of transcendence. Thus, it appears evident that character strengths and personality traits overlap but are not redundant, also adding incremental validity in, for instance, predicting life satisfaction (Park et al., 2004; West, 2006).

Moreover, occupational preferences and choices can also be ascribed to other sorts of dispositions than virtues and character strengths (e.g., interests, abilities, skills). For example, studies using the ‘Strong-Campbell Vocational Interest Inventory’ (Campbell, 1977) or being based on the ‘RIASEC typology of careers’ (realistic - investigative - artistic - social - enterprising - conventional; Holland, 1997) have investigated occupational preferences, medical careers, and chosen specialties. One study identified all medical disciplines to be throughout ‘investigative-social’ (Borges et al., 2004), whereas another study by Petrides and McManus (2004) revealed that e.g., surgery is rather a ‘realistic’ discipline (including people who like to work with things: here hands and tools, needing high levels of technical proficiency, craftsmanship and practical skills), internal medicine can be more assigned to the ‘investigative’ category (including people who like to work with data: exploring symptoms and relating them to latent causes to make a diagnosis), and psychiatry was considered to be more ‘artistic’ (including people who like to work with ideas: interpreting patients’ problems using various bio-psycho-social theories and responding individually to each patient). In turn, physicians who selected specialties with more pronounced social features also had higher scores on the Big Five dimension of ‘agreeableness,’ whereas higher ‘neuroticism’ implied rather a preference for ‘artistic’ and an aversion for ‘realistic’ or ‘enterprising’ specialties (Woods et al., 2016). Overall, two meta-analyses found three moderate relationships between personality traits (Big Five) and vocational interests (RIASEC) of medical students (see Duffy et al., 2009): ‘extraversion’ with ‘enterprising’ and ‘social,’ and ‘openness to experience’ with ‘artistic.’ However, character strengths as positively valued aspects of personality have hardly been related to the medical vocation before. In summary, the principle idea is that awareness of one’s individual character strengths may increase well-being and positivity, promote self-awareness on possible career paths, and improve workplace productivity and relationships. Nonetheless, there might be an accumulation of specific character strengths within certain professions, like ‘typical’ character strengths due to common life circumstances, experiences, study conditions or job specifications.

In the following, an overview of the most important findings of the conducted literature research will be presented. The searching strategy included the following terms: ‘character strengths’ or ‘values in action’ or ‘virtues’ and ‘medical students’ or instead of the students ‘medical doctors’ or ‘physicians’ or ‘resident physicians.’ The literature research was conducted in the following databases: APA Psycinfo, APA Psycarticles, Psyndex, Web of Science (Core Collection), Socindex, Pubmed/Medline, and Eric. In total, 160 hits revealed for medical students (time frame: 1971-2020) and 626 for physicians (time frame: 1816-2020). After screening all results, matching the search key with regards to content and considering double hits as well as multiple articles reporting on the same data, 43 peer-reviewed papers for medical students and 81 for physicians remained.

### Medical Students

Relating to virtues of medical students in general, most findings referred to (achieving) professionalism, virtuous caring, and good physicianhood. All these qualities overlap with Edmund Pellegrino’s proposed fundamental virtues of the medical profession, namely *benevolence, courage, compassion, fidelity to trust, intellectual honesty*, and *truthfulness* (Pellegrino, 2002). This prominent bioethicist pled for their tuition in medical school from the very beginning alongside knowledge and skills (Jacobson et al., 2006; Buyx et al., 2008; O’Sullivan and Toohey, 2008; Wear and Zarconi, 2008; Behrens and Fellingham, 2014; Magalhães-Sant’Ana, 2015; Shepherd et al., 2018). Therefore, the ‘Accreditation Council for Graduate Medical Education’ embedded respective virtues into graduate medical education in the late 1990s (overview in Larkin et al., 2005) as teaching professionalism and developing a good character can be understood as educators’ responsibility (Sehiralti et al., 2010; Carey et al., 2015). The ‘explicit’ professionalism curriculum puts patients into the center and supports altruistic attitudes, but the ‘implicit’ or ‘hidden’ curriculum that is defined by the learning environment in which it takes place (Hafferty and Franks, 1994) is often contrary, e.g., educators teaching opposite values by valuing appearance, formality, and conformity wrongly as ‘professional’ (Brainard and Brislen, 2007; Karches and Sulmasy, 2016). However, medical students’ altruistic behavior and empathy seem to be susceptible (Schweller et al., 2017; Sanjai and Gopichandran, 2018) and should be fostered by respective early curricular interventions during medical education.

Relating to character strengths of medical students in terms of the VIA-classification (Peterson and Seligman, 2004), eight empirical studies could be identified. Five studies derived from the ‘WELL-MED’ project (see section ‘Participants and Procedure’ for details) with two focusing on the applicability of character strengths and their associations with health-related outcomes (Hausler et al., 2017a; Huber et al., 2020), one illuminating the correlations of character strengths and different well-being aspects (Hausler et al., 2017b), one examining the development of cynicism (Kachel et al., 2020a), and one validating the VIA-120 short form (Höfer et al., 2019). However, none of these studies focused on identifying a certain profile of medical students. Thus, empirical data from this study will pursue this question. The top five character strengths of the medical students from this German-speaking sample were *fairness, honesty, judgment, kindness*, and *love*. In total, 19 character strengths met the criteria for at least a slight possession (see Harzer and Ruch, 2013). When asking British medical students to identify and rank the VIA-character strengths that they think best represent (a) their personal character and what they think (b) a good doctor would need, the most frequently answers were: (a) *fairness, honesty, kindness, perseverance* and *teamwork*, and (b) *fairness, honesty, judgment, kindness, leadership* and *teamwork* (Kotzee et al., 2017). In another study (Jones, 2013), British final year medical students were asked: ‘What are the most important character strengths of a good doctor?’. This study revealed *honesty* as the leading character strength, followed by *teamwork, judgment*, and *kindness* (descending order), whereas other frequently selected character strengths like *love of learning*, *perseverance*, or *social intelligence* were considered less important. Final year medical students in Oman rated as well (in descending order) *honesty, teamwork*, and *judgment* as being the most important VIA-character strengths for a physician, followed by *fairness, kindness*, and *love of learning* (Panambur et al., 2017). These six VIA-character strengths were also identified by them as most commonly observed in their teachers during the patient encounter. However, except for the ‘WELL-MED’ studies, participating medical students did not complete the VIA-questionnaire themselves revealing their own character strengths but they ranked the 24 character strengths from a descriptive list, respectively.

### Physicians

Most research on physicians’ virtues referred to professionalism accompanied with being a good doctor (also against cultural and/or spiritual backgrounds) and certain role virtues depending on medical specialty. Virtues have been already discussed in the early Stoic philosophy (e.g., Zeno’s four cardinal virtues: bravery, justice, temperance, and wisdom; Papadimos, 2004) and found their way into medical ethics through John Gregory (1724-1773) proposing compassion, integrity, self-effacement, and self-sacrifice to be essential for professionalism (Chervenak and McCullough, 2004). Modern clinical medicine and physician-patient relationships were significantly influenced by the book ‘The Virtues in Medical Practice’ by Pellegrino and Thomasa (1993; as cited in Fuks et al., 2012; Olivieri, 2018) addressing again the fundamental virtues (cf. above in ‘medical students’). Summarizing historical and modern literature, some virtues recur. In particular, compassion was discussed oftentimes to play a central role (e.g., Lopez and Dyck, 2009; Gelhaus, 2012; Aramesh, 2017) as well as courage (e.g., Shelp, 1984; Fugelli, 1999; Begley, 2008), altruism (e.g., Bishop and Rees, 2007), humility (e.g., Coulehan, 2011; DuBois et al., 2013), hope (e.g., Bryan, 2007; Miller, 2012), and practical wisdom (e.g., Corcoran et al., 2016; Bain, 2018). Professionalism in other countries or cultures is partially focusing on other values like in Korea, where physicians evaluated duties (e.g., responsibility, veracity) to be of higher importance than virtues (e.g., altruism; Kim and Choi, 2015). In Japan, rectitude was considered the most fundamental virtue (Nishigori et al., 2014) whereas in China benevolence and tolerance were important (Jing et al., 2013). Countries with a depressed economy emphasize a good understanding of medical ethics even more due to their prevailing economic situation, limited options of treatment, and cultural setting (Chukwuneke, 2015). Physicians’ different religions might also imply different (weighted) virtues, having consequently differing implications for treatment (e.g., Peteet, 2014; Gray, 2017).

Certain roles inherent to the medical profession (e.g., medical specialties, patient clientele) can ‘require’ certain virtues. Generally, in hospitals, physicians should be team players fulfilling all requirements for motivated and efficient employees (McDougall, 2013). For example in psychiatry, beneficence often conflicts with patients’ autonomy or needs (Kwok et al., 2012), where self-effacement could be particularly relevant in the case of prosecuting assaultive patients (Ho et al., 2009). When caring for so-called ‘difficult’ patients, again the virtues of courage and compassion were emphasized (Hawking et al., 2017). Beside technical skills, surgeons should cultivate practical wisdom (Hall, 2011) and humility (Toledo-Pereyra, 2007), and internists their integrity, respect, and compassion (Bergsma and Thomasa, 1985). Anesthesiologists are often confronted with pain and decision-making or palliative care, so they could particularly benefit from, for instance, justice, temperance, self-effacement, and wisdom according to literature (Diesfeld, 2008; Braun et al., 2010; Guevara-López et al., 2015; Kaldjian, 2019). However, today’s culture of medicine (example of the United States) is often hostile to ‘truthful’ professionalism and other qualities producing ‘good’ virtuous physicians as medicine has evolved into a giant, increasingly expensive technological profit center with young medical doctors only getting taught a list of required ‘professional’ practices (Coulehan, 2005).

Relating to character strengths of physicians in terms of the VIA-classification (Peterson and Seligman, 2004), eight empirical studies were identified. Six studies derived from the ‘WELL-MED’ project. Two of the six were using a combination of physicians’ and medical students’ data (Hausler et al., 2017a; Höfer et al., 2019), three focusing on character strengths’ applicability and (work-related) well-being in terms of (a) sociomoral climate (Höge et al., 2019), (b) work characteristics (Strecker et al., 2019), and (c) the distinction of character strengths’ application (Huber et al., 2019), and one following a mixed-methods design adding further insights into the relation of character strengths and physicians’ well-being (Kachel et al., 2020b). The latter article reports on opinions regarding the most important VIA-character strengths to feel well in the hospital. Resident physicians stated *social intelligence, teamwork, perseverance, fairness*, and *honesty* to be most important for well-being at work (descending order), whereas senior educators mentioned the character strength *humility* to be most relevant, followed by *teamwork, kindness, social intelligence*, and *zest*. However, none of these studies focused on identifying a certain physicians’ profile. The top five character strengths of the German-speaking hospital physicians from this sample were *fairness, honesty, judgment, kindness*, and *love*. Kotzee et al. (2017) asked British established doctors to identify and rank the VIA-character strengths that they think best represent their character and what they think a good doctor would need. There was a strong agreement between physicians and medical students concerning *fairness, honesty, kindness, perseverance* and *teamwork* representing their character, and that a good doctor is *fair, honest, kind*, a *leader*, a *good team player*, and a person with *good judgment*. Physicians reported to possess more *humor* than first-year undergraduates. Finally, in a Swiss physician sample, *love of learning* was the top character strength, followed by *curiosity, creativity, perseverance, perspective, honesty*, and *social intelligence* (Harzer, 2008), with *teamwork* in the last place. Beside the ‘WELL-MED’ studies, only data of the latter study revealed physicians’ prevalence of specific character strengths by answering the VIA-questionnaire whereas the others originated again from ranking all character strengths by description.

### Summary

Depending on the respective focus, different virtues or character strengths are desirable for medical students and physicians in the literature. The virtues of compassion, courage, altruism, and benevolence were found most often. Summarizing the VIA-classified character strengths, *fairness*, *honesty*, *kindness*, and *teamwork* were considered most often by respondents to be particularly important among both groups. Finally, according to the Declaration of Geneva, the modern-day physician’s pledge states explicitly to respect the patient’s autonomy and dignity, despite exercising beneficence and medical confidentiality toward the patients (Parsa-Parsi, 2017). Interestingly, increasing workload, occupational stress, and their potential adverse effects were considered as well in this pledge, leading to the intake of: ‘I will attend to my own health, wellbeing, and abilities in order to provide care of the highest standard.’ This clause reflects physicians’ humanity and their role of self-care being a part in improving patient care, but also offering more possibilities on character building among medical students and physicians due to its positive effect (e.g., Bryan and Babelay, 2009).

### Aims and Research Questions

The literature research revealed a majority of (a) theoretically conceptually driven papers and normative or philosophical research vs. empirical studies, and (b) ‘virtues’ in general with a striking plurality of different conceptions and theories vs. ‘character strengths’ in terms of the VIA-classification. Moreover, in previous studies (c) possible character strengths profiles have not been discussed so far also due to the lack of completed VIA-questionnaire data and (d) virtues as well as character strengths were hardly associated with well-being of medical students or physicians themselves but more with the question of ethics in patient care. Therefore, this study aims at adding empirical data concerning VIA-classified character strengths inherent in medical professionals (a/b) and giving evidence on possible profiles based upon valid questionnaire data with regards to their respective relevance and relation to well-being and work engagement (c/d). The following three exploratory research questions were addressed:

(I) What character strengths are the most prevalent in a sample of medical students and physicians giving evidence on a possible profile?
(II) Are there any differences in profiles of various sub-groups (e.g., different medical specialties)?
(III) How do character strengths of medical students and physicians relate to well-being and work engagement?

## Empirical Data

### Methods

#### Participants and Procedure

Data were collected within the ‘WELL-MED’ project from 2015 to 2020 at an Austrian medical university including two hospitals. In this longitudinal project, person- (e.g., character strengths) and condition-related (e.g., decision latitude, social support, cognitive demands) factors in terms of health and well-being of medical students and hospital physicians were investigated. With institutional review board approval, medical students (human medicine or dentistry) completed an annual online survey over a maximum period of six years, hospital physicians completed three surveys with a time lag of six months. A total of 584 baseline data sets were collected from medical students over the six year period. This sample consisted of 370 women (63.4%) and 214 men, the mean age was 20.8 ± 2.5 years (range: 21 to 38 years), and 55.7% Austrian, 19.9% German, and 19.3% Italian medical students participated. Longitudinal data (t1 - t2 - t3; time lag each one year) were available over a period of three years for 101 medical students. A total of 274 data sets were collected from hospital physicians. About 62% of them were female (*N* = 170) and the mean age was 34.2 ± 8.1 years (range = 24 to 64 years). A large majority (*N* = 224; 81.8%) were resident physicians in training, and 50 were senior medical specialists (18.2%). The physicians worked in 16 different medical disciplines. All participants completed the measurement of character strengths, 217 fully complete data sets were available for t1, 90 for t2 and 50 for t3.

### Measures

#### Character Strengths

Medical professionals’ character strengths were measured with the ‘Values in Action - Inventory of Strengths’ (VIA-IS; Peterson and Seligman, 2001; Peterson and Park, 2009). Höfer et al. (2019) validated the German short version consisting of 120-items in total. The 24 character strengths are rated on a five-point Likert scale ranging from 1 (very much unlike me) to 5 (very much like me). VIA-IS mean scores of 3.5 or higher are equal to possessing a character strength at least slightly (Harzer and Ruch, 2013). Item examples are: ‘I can always find the positive in what seems negative to others’ (hope), ‘I never quit a task before it is done’ (perseverance), or ‘Without exception, I support my teammates or fellow group members’ (teamwork). In this sample the internal consistency ranged from α = 0.63 (teamwork) to α = 0.91 (spirituality) for medical students, and from α = 0.61 (teamwork) to α = 0.90 (spirituality) for physicians.

#### Well-Being

General well-being (= thriving) was measured with the German version of the ‘Comprehensive Inventory of Thriving’ (CIT; Hausler et al., 2017). Thriving comprises 18 components, which can be summarized by seven subscales: subjective well-being (= SWB; life satisfaction, positive and negative feelings); relationship (support, community, trust, respect, loneliness, belonging), mastery (skills, learning, accomplishment, self-efficacy, self-worth), engagement, autonomy, meaning, and optimism. The latter six can be summarized to psychological well-being (PWB). The 54 items in total are rated on a five-point Likert scale ranging from 1 (strongly disagree) to 5 (strongly agree). Item examples are: ‘I am confident that I can deal with unexpected events’ (mastery), ‘There are people who appreciate me as a person’ (relationship), or ‘My life has a clear sense of purpose’ (meaning). Cronbach’s alpha for medical students as well as for physicians in this sample ranged from α = 0.95 (SWB) to α = 0.92 (PWB).

#### Work Engagement

Work engagement is defined as a fulfilling work-related positive state of mind and characterized by vigor, dedication and absorption (Schaufeli et al., 2006). To measure this construct, the German short version of the ‘Utrecht Work Engagement Scale’ (UWES; Schaufeli and Bakker, 2003; Schaufeli et al., 2006) was used with one version formulated for students and one for employees. Both consist of nine items, which are rated on a seven-point Likert scale ranging from 0 (never) to 6 (always). Item examples are: ‘My work inspires me’ or ‘At my study, I feel strong and vigorous’. Cronbach’s alpha was α = 0.94, for medical students as well as for physicians.

### Data Analysis

For all statistical analyses, SPSS Statistics 26 was used (IBM Corporation, 2018). Pearson’s coefficient inter-correlations can be interpreted with *r* < 0.10 = no correlation, *r* = 0.10−0.29 = low correlation, *r* = 0.30−0.49 = moderate correlation, *r* ≥ 0.50 = high correlation (Cohen, 1988). Acceptable internal consistency of an instrument is indicated by Cronbach’s α > 0.70 (see Peterson, 1994). T-tests were computed to compare baseline means of two groups (e.g., sex, training status), analyses of variance (ANOVAs) were applied to compare baseline means of multiple groups (e.g., medical specialties). The effect sizes regarding group differences will be represented as Cohen’s *d* (> 0.2 = small, > 0.5 = medium, > 0.8 = big; Cohen, 1988). Longitudinal regression analyses with all 24 character strengths as predictors were computed with thriving and work engagement as criterion (method: forward; last step mandatory including the criterion variable measured one year or six months before as control variable). Figures of character strengths profiles will not illustrate the whole possible scale spectrum of the VIA-IS (1-5) but a smaller range from 2 to 4.5 to improve readability.

### Results

#### Medical Students

##### (I) Character strengths prevalence

**FIGURE 1:**
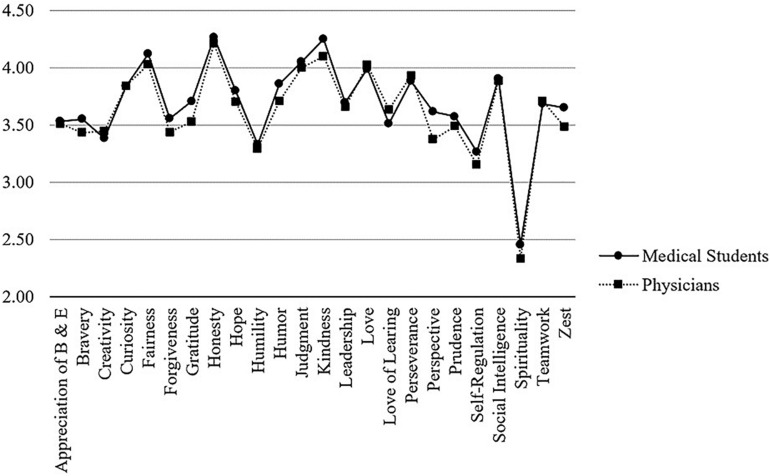
Comparison of VIA-character strengths between medical students and physicians.

Among the 584 medical students in this sample (completing t1), the VIA-character strength with the highest reported mean was *honesty* (*M* = 4.27, *SD* = 0.47), the lowest was *spirituality* (*M* = 2.45, *SD* = 1.04). Beside *honesty*, the five highest character strengths mean values (*M* ≥ 4.0) were found for *fairness*, *judgment, kindness*, and *love* (Table 1). Longitudinal data across three years revealed that these five character strengths remained on top with only little variation suggesting general stability. The order at t2 was identical, at t3, *honesty* and *kindness* changed the first and second place, and *judgment* and *love* the fourth and fifth place. These generally stable positioning trends recurred as well for the subsequent character strengths (e.g., 6th to 10th place). Furthermore, each of the top five character strengths significantly correlated with itself over time (*fairness: r* = 0.49−0.64, *honesty: r* = 0.54−0.58, *judgment: r* = 0.69−0.77, *kindness: r* = *0.61*−0.65, *love: r* = *0.61*−0.70; all *p* = 0.001). Figure 1 depicts the character strengths profile for the medical student sample.

**TABLE 1:** The 24 VIA-character strengths of medical students and physicians from the empirical data (t1).

VIA-character strengths	Rank	Mean	*SD*	Min	Max	Skewness	Kurtosis
	
	MS | P	MS | P	MS | P	MS | P	MS | P	MS | P	MS | P
Appreciation of Beauty and Excellence	19 | 15	3.53 | 3.51	0.74 | 0.66	1.0 | 1.6	5.0 | 5.0	−0.33 | −0.11	−0.04 | −0.50
Bravery	18 | 20	3.55 | 3.43	0.65 | 0.63	1.4 | 1.2	5.0 | 5.0	−0.08 | −0.15	−0.30 | 0.01
Creativity	21 | 18	3.38 | 3.45	0.72 | 0.69	1.2 | 1.0	5.0 | 5.0	0.02 | −0.25	−0.13 | 0.35
Curiosity	9 | 8	3.84 | 3.84	0.60 | 0.55	2.0 | 2.2	5.0 | 5.0	−0.37 | −0.37	−0.27 | 0.06
Fairness	3 | 3	4.12 | 4.03	0.57 | 0.55	1.8 | 2.0	5.0 | 5.0	−0.61 | −0.68	0.52 | 0.61
Forgiveness	17 | 19	3.56 | 3.44	0.64 | 0.63	1.0 | 1.8	5.0 | 5.0	−0.22 | −0.05	0.01 | −0.19
Gratitude	11 | 14	3.71 | 3.53	0.64 | 0.62	1.0 | 1.8	5.0 | 5.0	−0.34 | 0.07	0.30 | −0.25
Honesty	1 | 1	4.27 | 4.21	0.47 | 0.44	2.6 | 2.8	5.0 | 5.0	−0.48 | −0.27	−0.08 | −0.19
Hope	10 | 11	3.80 | 3.71	0.68 | 0.60	1.4 | 2.0	5.0 | 5.0	−0.54 | −0.31	0.14 | −0.01
Humility	22 | 22	3.33 | 3.29	0.67 | 0.63	1.6 | 1.4	5.0 | 4.8	−0.12 | −0.10	−0.16 | 0.01
Humor	8 | 10	3.86 | 3.71	0.71 | 0.68	1.6 | 2.0	5.0 | 5.0	−0.45 | −0.11	−0.14 | −0.37
Judgment	4 | 5	4.05 | 4.00	0.60 | 0.51	1.4 | 2.2	5.0 | 5.0	−0.58 | −0.20	0.38 | −0.09
Kindness	2 | 2	4.25 | 4.10	0.53 | 0.50	2.0 | 2.8	5.0 | 5.0	−0.61 | −0.18	0.38 | −0.27
Leadership	12 | 12	3.70 | 3.66	0.55 | 0.53	1.4 | 2.0	5.0 | 5.0	0.01 | 0.06	0.01 | −0.05
Love	5 | 4	3.99 | 4.03	0.67 | 0.67	1.2 | 1.6	5.0 | 5.0	−0.71 | −0.82	0.48 | 0.90
Love of Learning	20 | 13	3.51 | 3.64	0.74 | 0.68	1.6 | 1.6	5.0 | 5.0	−0.10 | −0.09	−0.65 | −0.29
Perseverance	7 | 6	3.88 | 3.93	0.65 | 0.58	1.8 | 2.0	5.0 | 5.0	−0.47 | −0.55	0.03 | 0.487
Perspective	15 | 21	3.62 | 3.37	0.62 | 0.53	2.0 | 2.0	5.0 | 5.0	−0.05 | 0.06	−0.40 | 0.21
Prudence	16 | 16	3.57 | 3.49	0.65 | 0.60	1.4 | 1.8	5.0 | 4.8	−0.35 | −0.16	−0.12 | −0.24
Self-Regulation	23 | 23	3.27 | 3.15	0.75 | 0.69	1.2 | 1.2	5.0 | 4.8	−0.04 | −0.19	−0.51 | −0.14
Social Intelligence	6 | 7	3.91 | 3.89	0.58 | 0.52	1.4 | 2.4	5.0 | 5.0	−0.49 | −0.30	0.59 | −0.08
Spirituality	24 | 24	2.45 | 2.33	1.04 | 0.95	1.0 | 1.0	5.0 | 5.0	0.54 | 0.56	−0.39 | −0.32
Teamwork	13 | 9	3.69 | 3.71	0.57| 0.50	1.4 | 2.0	5.0 | 5.0	−0.28| −0.31	0.52 | 0.54
Zest	14 | 17	3.65 | 3.49	0.66 | 0.65	1.6 | 1.8	5.0 | 5.0	−0.31 | −0.21	−0.08 | −0.25

*Note. MS, medical students (*N* = 584); P, physicians (*N* = 274); SD, Standard deviation.*

##### (II) Group differences

**FIGURE 2:**
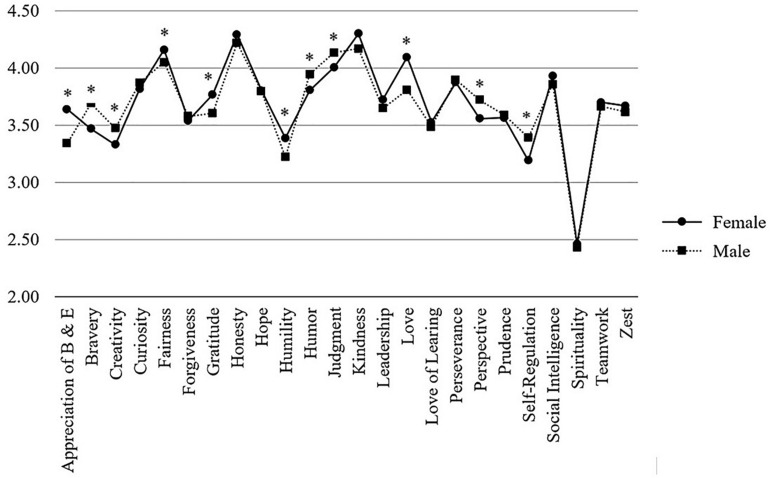
Comparison of VIA-character strengths between male and female medical students. *Note*. Character strengths marked with an asterisk are significantly different between groups.

Significant differences between female and male medical students were found for 11 character strengths. Women reported higher levels of *appreciation of beauty and excellence* (*M* = 3.64 vs. 3.34; *p* < 0.001), *fairness* (*M* = 4.16 vs. 4.05; *p* < 0.05), *gratitude* (*M* = 3.77 vs. 3.61; *p* < 0.01), *humility* (*M* = 3.39 vs. 3.22; *p* < 0.01), and *love* (*M* = 4.09 vs. 3.81; *p* < 0.001); men reported higher levels of *bravery* (*M* = 3.47 vs. 3.69; *p* < 0.01), *creativity* (*M* = 3.33 vs. 3.47; *p* < 0.05), *humor* (*M* = 3.81 vs. 3.95; *p* < 0.05), *judgment* (*M* = 4.01 vs. 4.14; *p* < 0.05), *perspective* (*M* = 3.56 vs. 3.72; *p* < 0.001), and *self-regulation* (*M* = 3.19 vs. 3.39; *p* < 0.001). However, all effect sizes were small (Cohen’s *d* ranging from 0.19 to 0.42). Character strength profiles of male and female medical students are displayed in Figure 2.

**FIGURE 3:**
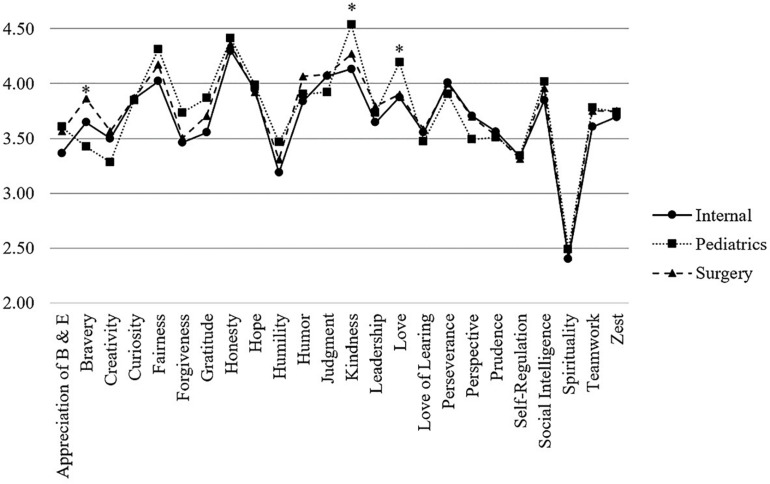
Medical students’ VIA-character strengths profile according to the preferred specialty. *Note*. Character strengths marked with an asterisk are significantly different between groups.

Medical students’ character strengths profiles were also compared regarding the three most desired future medical specialties students wanting to take up. Thus, their profiles were compared for the following groups: trauma and general surgery (*N* = 95), anesthesia, intensive care, and internal medicine (*N* = 47), and pediatric medicine (*N* = 48). According to ANOVA results, significant differences were found for *bravery* [*F*(2, 187) = 6.99, *p* = 0.001], *kindness* [*F*(2, 187) = 6.72, *p* < 0.002], and *love* [*F*(2, 187) = 3.28, *p* = 0.040]. Medical students being interested in pediatrics had higher mean values concerning *kindness* (*M* = 4.54) compared to those being interested in internal medicine (*M* = 4.13, *p* < 0.001, *d* = 0.84) or surgery (*M* = 4.27, *p* ≤ 0.05, *d* = 0.54), but they had lower mean values concerning *bravery* (*M* = 3.43) vs. medical students interested in surgery (*M* = 3.86; *p* < 0.001; *d* = 0.66). Concerning *love*, no further significant differences were evident according to the Bonferroni *post hoc* tests. The character strengths profiles for the three groups are pictured in Figure 3.

##### (III) Relation to well-being and work-engagement

The overall mean for thriving was *M* = 4.01 (*SD* = 0.43) and for work engagement *M* = 4.45 (*SD* = 0.90). Character strengths were positively related to overall well-being (thriving) and mostly to work engagement. *Judgment* and *humility* had low or no significant correlations with the well-being subscales, however, *spirituality* correlated significantly negatively with the subscale ‘autonomy’. *Forgiveness, humility* and *spirituality* did not significantly correlate with work engagement. In total, the strongest correlations with both outcomes were found for the character strengths *curiosity*, *gratitude*, *hope*, *love*, and *zest* (‘happiness strengths’). All correlation analyses of the VIA-character strengths and thriving with its seven subscales (SWB, relationship, engagement, mastery, autonomy, meaning, and optimism) as well as work engagement are shown in Table 2.

**TABLE 2:** Medical students’ correlations between VIA-character strengths and thriving with its seven subscales and work engagement (t1).

VIA-character strengths	CIT Categories							Thriving (general well-being)	Work Engagement
	Relationship	Engagement	Mastery	Autonomy	Meaning	Optimism	SWB		
Appreciation of Beauty and Excellence	0.17**	0.17**	0.22**	0.02	0.14**	0.16**	0.10*	0.21**	0.14**
Bravery	0.10*	0.22**	0.34**	0.03	0.22**	0.14**	0.12**	0.23**	0.21**
Creativity	0.11**	0.26**	0.36**	–0.03	0.11*	0.14**	0.11*	0.22**	0.30**
Curiosity	0.33**	0.46**	0.53**	0.10*	0.30**	0.37**	0.41**	0.50**	0.43**
Fairness	0.28**	0.22**	0.32**	0.11*	0.17**	0.19**	0.19**	0.31**	0.15**
Forgiveness	0.16**	0.11*	0.17**	0.02	0.11*	0.26**	0.18**	0.21**	0.06
Gratitude	0.34**	0.32**	0.39**	0.08	0.41**	0.38**	0.39**	0.46**	0.31**
Honesty	0.30**	0.25**	0.36**	0.19**	0.23**	0.18**	0.17**	0.34**	0.16**
Hope	0.42**	0.40**	0.56**	0.17**	0.58**	0.70**	0.65**	0.68**	0.42**
Humility	0.06	0.13**	0.11*	0.07	0.05	0.04	0.04	0.09*	–0.03
Humor	0.25**	0.31**	0.35**	0.03	0.22**	0.32**	0.34**	0.37**	0.25**
Judgment	0.06	0.06	0.31**	0.09*	0.12**	0.07	0.04	0.16**	0.21**
Kindness	0.32**	0.20**	0.30**	0.13**	0.17**	0.22**	0.20**	0.33**	0.19**
Leadership	0.29**	0.26**	0.37**	0.03	0.20**	0.19**	0.20**	0.33**	0.23**
Love	0.43**	0.25**	0.32**	0.13**	0.27**	0.38**	0.43**	0.47**	0.13**
Love of Learning	0.09*	0.20**	0.32**	0.11*	0.10*	0.10*	0.14**	0.21**	0.34**
Perseverance	0.24**	0.31**	0.45**	0.14**	0.36**	0.26**	0.24**	0.39**	0.29**
Perspective	0.08	0.13**	0.38**	0.09	0.17**	0.22**	0.17**	0.25**	0.21**
Prudence	0.10*	0.08	0.28**	0.11*	0.17**	0.13**	0.09*	0.19**	0.15**
Self-Regulation	0.16**	0.31**	0.27**	0.09*	0.18**	0.17**	0.21**	0.26**	0.22**
Social Intelligence	0.35**	0.23**	0.36**	0.12**	0.24**	0.28**	0.27**	0.39**	0.26**
Spirituality	0.21**	0.13**	0.13**	−0.11*	0.25**	0.19**	0.15**	0.20**	0.07
Teamwork	0.30**	0.23**	0.29**	0.06	0.19**	0.18**	0.21**	0.32**	0.19**
Zest	0.47**	0.56**	0.56**	0.07	0.45**	0.53**	0.56**	0.64**	0.48**

*p ≤ 0.05, **p ≤ 0.01.

Concerning the longitudinal regression analyses with all 24 character strengths as predictors, and thriving or work engagement as criterion, stepwise (forward) regression analyses revealed the following. Analyses (time lag one year) with thriving (t2) as criterion (*N* = 200) showed significant positive standardized regression effects for *curiosity* (β = 0.24, *t* = 2.63, *p* = 0.009) and *zest* (β = 0.37, *t* = 4.17, *p* < 0.001) on thriving, while negative effects were apparent for *appreciation of beauty and excellence* (β = −0.24, *t* = −3.30, *p* = 0.001) and *perspective* (β = −0.17, *t* = −2.13, *p* = 0.034). When controlled for thriving at t1 in a second step, *appreciation of beauty and excellence* (β = −0.18, *t* = −2.96, *p* = 0.003), *perspective* (β = −0.14, *t* = −2.05, *p* = 0.042), and *zest* (β = 0.21, *t* = 2.65, *p* = 0.009) remained significant. Regression analyses (*N* = 110) between character strengths (t2) and thriving (t3) showed one significant regression coefficient for *hope* (β = 0.34, t = 2.55, *p* = 0.013). When controlled for thriving at t2, no regression analysis remained significant.

Defining work engagement (t2) as criterion and character strengths as predictors (t1), analyses (*N* = 202) showed a negative significant standardized regression effect for *hope* (β = −0.17, *t* = −1.98, *p* = 0.049) and a positive one for *zest* (β = 0.42, *t* = 3.99, *p* < 0.001) on work engagement. When controlled for work engagement at t1 in a second step, *creativity* (β = −0.18, *t* = −2.28, *p* = 0.024), *hope* (β = −0.29, *t* = −3.69, *p* < 0.001), and *zest* (β = 0.30, *t* = 3.13, *p* = 0.002) appeared significant. Regression analyses examining character strengths (t2) and work engagement (t3; *N* = 111) revealed a significant effect for *self-regulation* (β = 0.28, *t* = 2.49, *p* = 0.015). When controlled for work engagement at t2, the regression analysis remained significant for *self-regulation* (β = 0.21, *t* = 2.30, *p* = 0.024).

#### Hospital Physicians

##### (I) Character strengths prevalence

The VIA-character strengths profile in the sample of the 274 hospital physicians (completing t1) resulted in the following. The highest mean value was reported for *honesty* (*M* = 4.21, *SD* = 0.44), and the lowest for *spirituality* (*M* = 2.33, *SD* = 0.95). Beside *honesty*, the top five character strengths in this sample (*M* ≥ 4.0) were *fairness*, *judgment, kindness*, and *love* (Table 1). Longitudinal data across three years revealed that these five character strengths remained in front as the top five strengths but with some variation. At t2, *love* moved one position forward as well as *judgment*, while *fairness* dropped slightly. At t3, *judgment* and *kindness* changed the fourth and second place compared to t2. These positioning trends recurred as well for the subsequent character strengths (e.g., 6th to 10th place) suggesting overall general stability. Furthermore, each of the top five character strengths significantly correlated with itself over time (*fairness: r* = 0.68 −0.82, *honesty: r* = 0.62 −0.80, *judgment: r* = 0.71 −0.75, *kindness: r* = *0.69* −0.82, *love: r* = *0.80* −0.86; all *p* = 0.001). Figure 1 displays the profile for this sample.

##### (II) Group differences

**FIGURE 4:**
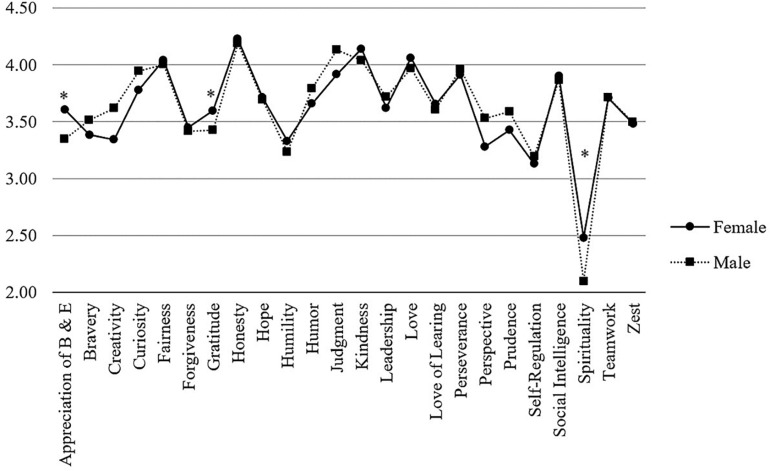
Comparison of VIA-character strengths between male and female physicians. *Note*. Character strengths marked with an asterisk are significantly different between groups.

Looking at the differences between character strengths profiles of female and male hospital physicians, results showed an overall homogeneous picture (Figure 4). Significant differences appeared for women reporting higher levels of *appreciation of beauty and excellence* (*M* = 3.61 vs. 3.35, *p* < 0.01), *gratitude* (*M* = 3.60 vs. 3.43, *p* < 0.05), and *spirituality* (*M* = 2.48 vs. 2.10, *p* < 0.01). On the other hand, men rated themselves significantly higher in terms of *creativity* (*M* = 3.62 vs. 3.34, *p* < 0.01), *curiosity* (*M* = 3.94 vs. 3.78, *p* < 0.05), *judgment* (*M* = 4.13 vs. 3.92, *p* < 0.01), *perspective* (*M* = 3.53 vs. 3.28, *p* < 0.001), and *prudence* (*M* = 3.59 vs. 3.43, *p* < 0.05). However, all effect sizes were small (Cohen’s *d* ranging from 0.27 to 0.49).

**FIGURE 5:**
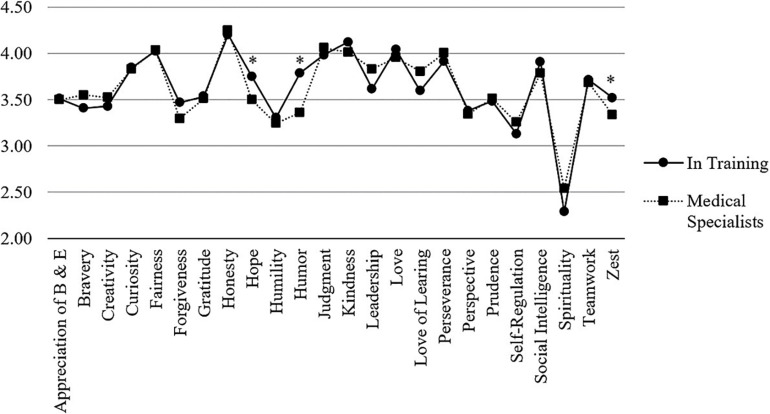
Comparison of VIA-character strengths between physicians in training and medical specialists. *Note*. Character strengths marked with an asterisk are significantly different between groups.

Physicians’ character strengths profiles were also analyzed regarding their training status which also strongly and naturally correlated with age (*r* = 0.66, *p* < 0.001). The overall picture resulted in a quite homogenous one (Figure 5). Nevertheless, physicians in training scored significantly higher in terms of *hope* (*M* = 3.75 vs. 3.50, *p* < 0.01, *d* = 0.45), *humor* (*M* = 3.79 vs. 3.36, *p* < 0.001, *d* = 0.64), and *zest* (*M* = 3.52 vs. 3.34, *p* < 0.05, *d* = 0.29), whereas medical specialists scored significantly (*p* < 0.05) higher in *leadership* (*M* = 3.83 vs. 3.62, *d* = 0.38) and *love of learning* (*M* = 3.81 vs. 3.60, *d* = 0.33).

**FIGURE 6:**
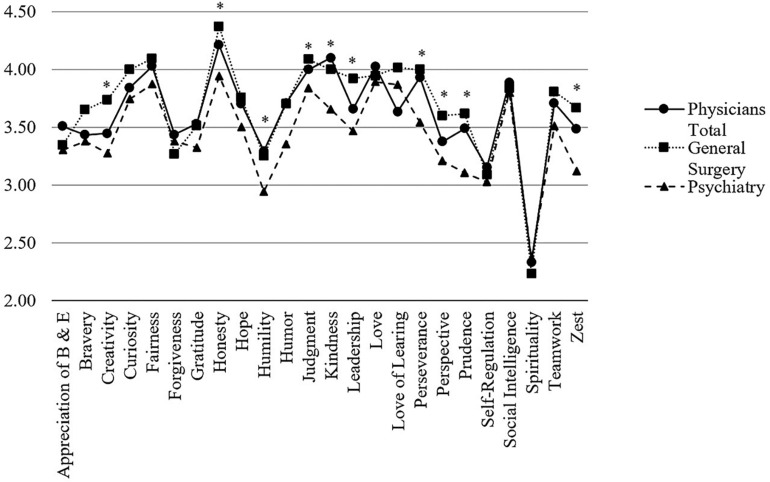
Comparison of VIA-character strengths between physicians of different specialties. *Note*. Character strengths marked with an asterisk are significantly different between groups.

Comparing character strengths profiles of different medical disciplines revealed a general tendency toward the same picture for all hospital physicians in this sample. The focus was on medical disciplines comprising 20 participants or more. The largest group were physicians with a specialization in anesthesiology (*N* = 50), followed by general surgery (*N* = 23), psychiatry (*N* = 21), and internal medicine (*N* = 20). Significant mean differences were found in 10 of the 24 character strengths and were most often evident when comparing general surgery and psychiatry. Those two groups are depicted in Figure 6, whereas the profiles of anesthesiologists and internal medicals (as they are almost identical to the profile of the physicians in total) will not be depicted for better readability. All results refer mainly to aspiring medical specialists.

According to ANOVA results with Bonferroni *post hoc* analyses, anesthesiologists - compared to general surgeons - had significantly lower mean values of *leadership* [*F*(3, 110) = 3.08, *p* < 0.05; *M* = 3.55 vs. *M* = 3.92, *p* < 0.05, *d* = 0.52] and *perspective* [*F*(3, 110) = 3.42, *p* < 0.05; *M* = 3.23 vs. *M* = 3.60, *p* < 0.05, *d* = 0.65], whereas they had - compared to psychiatrists - significantly higher mean values of *honesty* [*F*(3, 110) = 5.06, *p* < 0.01; *M* = 4.24 vs. *M* = 3.94, *p* < 0.01, *d* = 0.71] and *kindness* [*F*(3, 110) = 4.75, *p* < 0.01; *M* = 4.18 vs. *M* = 3.66, *p* < 0.05, *d* = 1.13]. Internal medicals - compared to psychiatrists - reported significant higher mean values of *honesty* [*F*(3, 110) = 5.06, *p* < 0.01; *M* = 4.32 vs. *M* = 3.94, *p* < 0.01, *d* = 0.95], *humility* [*F*(3, 110) = 2.81, *p* < 0.05; *M* = 3.47 vs. *M* = 2.94, *p* < 0.05, *d* = 0.95] and *judgment* [*F*(3, 110) = 3.09, *p* < 0.05; *M* = 4.25 vs. *M* = 3.84, *p* < 0.05, *d* = 0.93]. General surgeons had - compared to psychiatrists - significantly higher mean values of *honesty* [*F*(3, 110) = 5.06, *p* < 0.01; *M* = 4.37 vs. *M* = 3.94, *p* < 0.01, *d* = 1.02], *leadership* [*F*(3, 110) = 3.08, *p* < 0.05; *M* = 3.92 vs. *M* = 3.47, *p* < 0.05, *d* = 0.82], *prudence* [*F*(3, 110) = 3.49, *p* < 0.05; *M* = 3.62 vs. *M* = 3.11, *p* < 0.05, *d* = 1.19], *perseverance* [*F*(3, 110) = 2.89, *p* < 0.05; *M* = 4.00 vs. *M* = 3.54, *p* = 0.05, *d* = 0.88], and *zest* [*F*(3, 110) = 3.24, *p* < 0.05; *M* = 3.67 vs. *M* = 3.12, *p* < 0.05, *d* = 0.86]. All differences between medical specialties can be found in the Supplementary Material.

##### (III) Relation to well-being and work-engagement

The overall mean for thriving was *M* = 3.85 (*SD* = 0.41) and for work engagement *M* = 3.62 (*SD* = 1.08). Mostly, character strengths were positively related to overall well-being (thriving) and work engagement. *Humility* and *spirituality* showed no significant correlations with the well-being subscales, and *spirituality* was significantly negatively correlated with the subscale ‘autonomy’. *Appreciation of beauty, fairness, forgiveness, humility, kindness*, and *spirituality* did not significantly correlate with work engagement. Again, the ‘happiness strengths’ showed highest correlations to most well-being subscales, while work engagement correlated the most with *curiosity*, *hope*, *love of learning*, and *zest.* All correlation analyses of the VIA-character strengths and thriving with its seven subscales (SWB, relationship, engagement, mastery, autonomy, meaning, and optimism) as well as work engagement are shown in Table 3.

**TABLE 3:** Physicians’ correlations between VIA-character strengths and thriving with its seven subscales and work engagement (t1).

VIA-character strengths	CIT Categories							Thriving (general well-being)	Work Engagement
	Relationship	Engagement	Mastery	Autonomy	Meaning	Optimism	SWB		
Appreciation of Beauty and Excellence	0.17*	0.19**	0.13	–0.09	0.12	0.14*	0.15*	0.17**	0.06
Bravery	0.08	0.28**	0.23**	0.02	0.14*	0.03	–0.02	0.13	0.19**
Creativity	0.06	0.28**	0.34**	0.04	0.15*	0.04	0.01	0.17*	0.21**
Curiosity	0.28**	0.52**	0.45**	0.18**	0.34**	0.45**	0.38**	0.48**	0.35**
Fairness	0.25**	0.27**	0.11	0.04	0.18**	0.14*	0.16*	0.22**	0.10
Forgiveness	0.20**	0.20**	0.14*	0.10	0.09	0.19**	0.18**	0.22**	0.10
Gratitude	0.40**	0.29**	0.23**	0.00	0.31**	0.28**	0.31**	0.38**	0.19**
Honesty	0.32**	0.25**	0.23**	0.13	0.31**	0.15*	0.24**	0.33**	0.14*
Hope	0.40**	0.58**	0.55**	0.24**	0.57**	0.64**	0.63**	0.67**	0.48**
Humility	0.10	–0.08	0.05	–0.07	0.09	–0.10	–0.05	0.02	0.03
Humor	0.12	0.27**	0.28**	–0.02	0.10	0.31**	0.23**	0.25**	0.24**
Judgment	0.06	0.18**	0.28**	0.12	0.08	0.03	0.06	0.16*	0.14*
Kindness	0.26**	0.24**	0.20**	–0.05	0.15*	0.17*	0.15*	0.24**	0.13
Leadership	0.15**	0.19**	0.28**	0.04	0.23**	0.12	0.08	0.21**	0.15*
Love	0.49**	0.30**	0.31**	0.16*	0.39**	0.36**	0.42**	0.50**	0.17*
Love of Learning	0.14*	0.38**	0.30**	0.15*	0.21**	0.20**	0.21**	0.28**	0.33**
Perseverance	0.21**	0.28**	0.27**	0.18**	0.34**	0.12	0.17*	0.29**	0.20**
Perspective	0.09	0.25**	0.34**	–0.07	0.15*	0.20**	0.16*	0.23**	0.26**
Prudence	0.15*	0.12	0.23**	0.09	0.19**	0.10	0.13	0.21**	0.19**
Self-Regulation	0.16*	0.14	0.12	0.00	0.17*	0.09	0.13	0.17*	0.15*
Social Intelligence	0.19**	0.24**	0.28**	0.02	0.15**	0.23**	0.16*	0.25**	0.17*
Spirituality	0.15*	–0.07	–0.04	−0.21**	0.05	–0.06	–0.03	0.01	–0.01
Teamwork	0.29**	0.26**	0.28**	0.10	0.26**	0.21**	0.24**	0.33**	0.22**
Zest	0.40**	0.65**	0.52**	0.17*	0.48**	0.60**	0.59**	0.64**	0.67**

*p ≤ 0.05, **p ≤ 0.01.

Concerning the longitudinal regression analyses with all 24 character strengths as predictors, and thriving or work engagement as criterion, stepwise (forward) regression analyses revealed the following. Analyses (time lag 6 months) with thriving (t2) as criterion (*N* = 88) showed significant standardized regression effects for *hope* (β = 0.64, *t* = 8.18, *p* < 0.001) and *love* (β = 0.21, *t* = 2.66, *p* < 0.01) on thriving. When controlled for thriving at t1 in a second step, *hope* (β = 0.24, *t* = 2.50, *p* < 0.05) remained significant. Regression analyses (*N* = 50) between character strengths (t2) and thriving (t3) showed one significant regression coefficient for *hope* (β = 0.69, *t* = 6.55, *p* < 0.001), but when controlled for thriving at t2, no significant path remained.

Defining work engagement (t2) as criterion and character strengths as predictors (t1), analyses (*N* = 90) showed significant standardized regression effects over six months for *teamwork* (β = 0.19, *t* = 2.12, *p* < 0.05) and *zest* (β = 0.52, *t* = 5.90, *p* < 0.001) on work engagement. When controlled for work engagement at t1 in a second step, only the control variable remained significant. Performing the same analyses between t2 and t3 (*N* = 50), significant standardized regression effects over six months revealed for *perseverance* (β = 0.29, *t* = 2.57, *p* < 0.05), *zest* (β = 0.55, *t* = 5.06, *p* < 0.001), and a negative effect for *bravery* (β = −0.23, *t* = −2.10, *p* < 0.05) on work engagement. When controlling for work engagement at t2, a significant path for *zest* (β = 0.34, *t* = 2.06, *p* < 0.05) remained while the control variable was not significant for the first time.

## Discussion

This paper reported on known evidence of virtues and VIA-character strengths for medical students and physicians. Literature showed that depending on the respective focus or research area, different virtues and character strengths were evident. Based on the few empirical studies using the VIA-classification of character strengths (Peterson and Seligman, 2004), *fairness*, *honesty*, *judgment, kindness*, and *love* were reported to have the highest means in medical professionals, even though these results have to be attributed predominantly to the ‘WELL-MED’ studies. *Honesty, fairness*, and *kindness* together with *teamwork* were consistently rated by medical professionals to be important for being a good doctor. In this study, differences between medical specialties revealed the biggest effect sizes, with psychiatrists consistently reporting lower character strength means. The ‘happiness strengths’ *curiosity*, *gratitude*, *hope*, *love*, and *zest* consistently had the highest correlations with thriving cross-sectionally. Long-term results of character strengths influencing well-being and work engagement revealed positive effects (*perseverance, self-regulation, teamwork, zest*), negative effects (*appreciation of beauty and excellence, bravery, creativity*, *perspective*) or even both (*hope*).

Addressing the first two research questions (I and II), medical students and physicians differed only a little in terms of (1) sex and (2) training status, whereas considerable differences were found regarding their (3) aspired medical specialty.

(1) Female medical students and physicians reported significantly higher values of *appreciation of beauty and excellence.* Persons with this character strength notice and appreciate beauty, excellence and/or skilled performance in all domains of life, from nature to art to mathematics to science to everyday experience (Seligman, 2002). Other studies found women in general to be more amenable to a conscious perception of beautiful things valuing them (e.g., Ovejero and Cardenal, 2011; Littman-Ovadia and Lavy, 2012). Higher scores regarding *love* (medical students) and *gratitude* (both samples) were also shown for women in the two cited studies before, whereas men reported on more *creativity* (cf. Linley et al., 2007), *judgment*, and *perspective* in both samples. Masculine norms (e.g., primacy of work, or pursuit of status) seem to be particularly contrary to *appreciation of beauty and excellence* beside the fact that men in general reported lower character strengths scores while they tended to increase with comfort to feminine norms (Ovejero and Cardenal, 2011). Socialization processes and gender roles determine the degree to which women and men prioritize morality and experience morally relevant emotions (Ward and King, 2018). Women are expected to be caring and warm (in line with the caregiving role), to consider morality and kindness as integral parts, and to experience negative emotions when people violate the community’s welfare. This imprint might also explain the higher means in terms of VIA-character strengths being morally valued traits. However, all effect sizes were consistently small.

(2) Physicians in training reported in particular significantly higher values of *humor* compared to medical specialists. In the VIA-definition, the scope of *humor* is intentionally restricted only to forms that serve some moral good, e.g., offering the lighter side to others, making others smile or laugh, building social bonds and lubricating social interaction, or coping with stressful situations. But also other forms of humor exist with some of them being aggressive, self-defeating or clearly mean (e.g., mockery, ridicule, sarcasm) or at the border (e.g., parody, practical jokes; Müller and Ruch, 2011). Possibly, this ‘socially warm’ humor style pictured by the VIA-IS, is more prevalent in physicians in training as there is more support, solidarity, and collaboration among them and colleagues building social bonds, whereas medical specialists perceive more competition and work mostly on their own. Thus, physicians in training have more possibilities to cultivate relationships with others. Working over years in a hierarchical system with high strain and decreasing valuable social interactions could possibly lead to an increase or change toward other forms of ‘humor’ not represented in the VIA-IS, e.g., sarcasm or cynicism. Contrarily, one cited study showed that British doctors (with at least five years of experience) reported to possess more *humor* than medical students (Kotzee et al., 2017). However, when taking a closer look at the sample, only one fifth of them were hospital physicians (vs. general and other private practitioners) possibly supporting the aforementioned assumption that hospitals could re-weigh individuals’ character strength toward a different type of humor.

(3) Medical students being interested in pediatrics reported significantly higher means regarding *kindness* than students interested in surgery or internal medicine. This result makes sense as working with children particularly requires the ability to be caring, supportive, and compassionate with a deep concern for the little patients’ welfare. Students being interested in surgery reported more *bravery* than ongoing pediatrists. This result is consistent with two cited studies where neuroticism (i.e., inhibition, shyness, emotional lability) negatively predicted *bravery* and surgery was considered to be a more ‘realistic’ discipline (Petrides and McManus, 2004; Macdonald et al., 2008), implying that surgeons tackle problems, face medical challenges, and react quickly considering immediate consequences. In general, (aspiring) surgeons in this sample reported throughout the highest VIA-means across many character strengths, in particular compared to psychiatrists who rated themselves continuously lower among all character strengths.

Today’s medical culture teaches young physicians to develop self-confidence quickly and to move beyond all insecurities. Surgeons might be affected by this issue in particular, as they first have to cause the patient some harm to achieve a benefit for them (e.g., trying saving lives). This might sometimes lead to exaggerated levels of self-confidence and reduced self-reflection, illustrated e.g., by a patient’s statement who said he could always tell when surgeons enter the room: ‘You enter with an air of bravado and arrogance that the medical doctors do not exude’ (Angelos, 2017). Surgeons in this sample rated all character strengths comparatively high, including the character strengths of virtue ‘wisdom’ (*creativity, curiosity, judgment, love of learning*, *perspective*) and *humility*. This raises the question whether the scores were influenced by increased levels of self-confidence or if they are taking up the idea previously described by Hall (2011) and Toledo-Pereyra (2007) to cultivate practical wisdom or humility beside technical skills. The biggest difference compared to psychiatrists was found for *prudence* (being careful about one’s choices, thinking before acting, involving far-sighted and short-term planning) which is also often referred to as practical wisdom. Obviously, surgeons can cause greater physical, observable, and in the worst case lethal harm when their treatment fails (leading to higher means of *prudence*). In contrast, psychiatrists treat patients with mental, emotional, and behavioral disorders by developing treatment plans, prescribing medication, conducting conversations, and applying therapeutic interventions. They should have strong listening skills, be perceptive, reflective, and able to provide crisis intervention when needed as their patients cannot be ‘cured by scalpel.’ Therefore, their way of treating patients is fundamentally different based upon a more holistic (e.g., bio-psycho-social) view on persons’ health and disease with many chronic patients consulting them again and again. This interpretation of their working style is consistent with some cited studies that considered psychiatry to be rather ‘artistic’ which is in turn positively associated with ‘openness to experience’ but also with ‘neuroticism’ (Petrides and McManus, 2004; Duffy et al., 2009; Woods et al., 2016). However, as this discipline is not as straightforward or concrete as surgery, this might mislead to the assumption of less ‘impressive’ work, receiving further support from the hospital when paged for patients on the somatic wards for only prescribing psychotropics. Their remaining knowledge or treatment repertoire is oftentimes not asked, conveying little appreciation and a poorer reputation. According to literature, psychiatrists’ benevolence often conflicts with patients’ autonomy and their self-effacement is relevant (Ho et al., 2009; Kwok et al., 2012). All character strengths can be interpreted as beneficial due to their definition and therefore, possibly striking psychiatrists as being generally ‘inappropriate’ within their work. Moreover, if *honesty* (i.e., speaking the truth, presenting oneself and one’s reactions genuinely to each person) is understood as the ‘opposite’ of self-effacement implying to show all internal feelings, intentions, and commitments unfiltered even in precarious situations, the big difference concerning *honesty* compared to all other medical specialties in this sample would be traceable, as therapists (psychiatrists) should be discreet in sharing honest appraisals with the patient (Salzman, 1973). Therefore, taking all these points together, psychiatrists might remain self-effacing in terms of all character strengths and rate them lower.

Addressing the third research question (III), positive effects of character strengths on well-being have already been demonstrated (e.g., Peterson and Park, 2006; Seligman, 2011) but not many studies illuminated what aspects of well-being are influenced. In this study, various aspects of thriving were cross-sectionally analyzed showing that in both samples *humility* was mostly not associated with any aspect of thriving and neither were *judgment* (students) and *spirituality* (physicians). The latter even had a clearly negative relation with ‘autonomy’ (control) in both samples. *Spirituality* comprises many different aspects, e.g., life calling, beliefs about the universe, and practices that connect with the transcendent (‘sacred’) which is blessed, holy, or particularly special (secular or non-secular). It involves the belief that there is a dimension to life beyond human understanding being in contrast to ‘autonomy’ defined by life decisions on one’s own responsibility, belief in one’s personal skills, and internal locus of control. In both samples, *humor* was positively associated with ‘optimism,’ and *perseverance* with ‘meaning’. In total, ‘mastery’ (skills, learning, accomplishment, self-efficacy, and self-worth) was clearly linked to most of the character strengths. In both samples, *love of learning* was explicitly associated with work engagement.

Longitudinal data examining possible effects of character strengths on later well-being and work engagement revealed significant results for (1) medical students’ *appreciation of beauty and excellence, creativity*, *hope, perspective, self-regulation*, and *zest*, and (2) physicians’ *bravery*, *hope*, *perseverance*, and *teamwork.*

(1) Initial *zest* led to positive effects on medical students’ well-being one year later whereas *appreciation of beauty and excellence* and *perspective* seemed to have a negative impact. *Zest* implies approaching situations fully tilted with excitement and energy, i.e., being enthusiastic despite all the new demands and strains at the beginning of a medical study. On the other hand, particularly in the first year there is neither the time nor the need (or institutional calling) to recognize, experience, and *appreciate beauty* around one or others’ skills, potentially frustrating students who set a high value on this. Moreover, studying medicine is possibly not that ‘beautiful’ or ‘excellent’ as the aspired job afterward, leading to well-being decreases. *Perspective* (i.e., to think in big terms and avoid getting wrapped up in small details when there are bigger issues to consider) follows the same trajectory in terms of frustration as there are far too many small things at the beginning of a medical study to organize requiring full attention while the bigger picture (e.g., finally becoming a physician) has taken a back seat. Regarding future work engagement, initial *creativity*, *hope*, and *zest* were relevant for outcomes after one year, whereas *self-regulation* was rather important in the third year. Interestingly, initial *creativity* and *hope* influenced work engagement negatively. In medical school, everything is thoroughly structured and planned in the first year following a tight schedule. They have to learn physiology, biochemistry, anatomy, etc. where ‘*creative*’ ideas or perspectives might be not asked or even obstructively. Furthermore, first year medical students experience much external control by the institution, educators and examinants contrasting with their *hope* (e.g., confidence that goals can be reached effectively by one’s own agency), leading to less self-efficacy and involvement with working tasks. With increasing demands and strains over time, *self-regulation* gained relevance for third years’ work engagement. This character strength is complex (i.e., regulating one’s actions, controlling one’s emotions and reactions to disappointment or insecurities) but was also associated with higher ‘agreeableness’ (e.g., Haslam et al., 2004) including the sub-trait of ‘compliance’ meaning that one does what one is required or expected to do. This is in line with the finding here, as both support the ability to keep a sense of order and progress in life helping to stay involved with ‘work’.

(2) Physicians’ longitudinal data revealed that *hope* had positive effects on their future well-being across all measurement time points. Beside the belief that many effective pathways can be devised in order to get to that desired goal, having positive expectations about the future is inherent to *hope*. This optimistic thinking can be interpreted as part of well-being in terms of optimism (Scheier and Carver, 1985) also included in the CIT. Physicians’ future work engagement was clearly predicted by *zest* across all time points. As their definitions highly overlap in terms of content (both including excitement, dynamics, and energy with approaching tasks not halfheartedly), other character strengths might provide more information, like *bravery*, *perseverance*, or *teamwork.* In particular, at the beginning of a medical career, *teamwork* seemed relevant for ensuing work engagement, whereas *perseverance* was more important in the further course to stay engaged, in contrast to *bravery*, which had a negative impact on work engagement in the third year. In this context, *bravery* might have been understood as fulfilling the demand to hang on or withstand physicians’ adverse working conditions. This strategy could possibly be useful for a short period of time in terms of exploiting oneself toward this requirement but then turning into decreased work engagement with just persevering in the circumstances. When remembering the study of Macdonald et al. (2008) it is quite interesting that the Big Five dimension ‘neuroticism’ negatively predicted *bravery* and *hope*, both having an impact here on physicians’ future well-being and work engagement. However, as these effects were directed differently, the role of possible underlying ‘neuroticism’ is not clear needing further evidence (Macdonald et al., 2008).

Summarizing, character strengths profiles differed in parts for medical specialties, in particular for general surgeons and psychiatrists with biggest effects for *honest*y and *prudence*. The top five character strengths were not influential on long-term well-being or work engagement of medical professionals, instead *hop*e, *perseverance, self-regulation*, *teamwork* or *zest* showed more influence over time. Possessing these character strengths alone might not be enough to derive well-being benefits. Applying those character strengths to foster deepened positive experience may be more relevant to increase well-being (e.g., Govindji and Linley, 2007; Littman-Ovadia and Steger, 2010; Seligman, 2011; Harzer and Ruch, 2012, 2013). One study supports the assumption that the possession as well as the applicability of signature character strengths at work and in private life is important but to different degrees, also depending on the respective outcome (Huber et al., 2019). However, that study considered cross-sectional data only. Therefore, deriving long-term well-being or work engagement effects might rather require the application of character strengths.

The question arises whether the top five character strengths in this sample are specific for medical professionals or if their profile is similar compared to other German-speaking samples or socially oriented occupational groups. Ruch et al. (2010) validated the German VIA-IS (240 items) in a Swiss general population sample (*N* = 1674) with *curiosity, fairness, kindness, honesty, and love* having the highest means. By investigating women over time, Proyer et al. (2011) found *curiosity, love of learning, love, kindness, and fairness* to be the top five in Switzerland (*N* = 1087). A study validating the German shorter VIA-IS form with 120 items in a representative sample (Höfer et al., 2019), revealed the highest means for *honesty, kindness, fairness, perseverance*, and *love* representing the general German population (*N* = 1073). Results from the VIA-240 in the latter study showed *fairness, honesty, kindness, curiosity, humor*, and *judgment* in front. Therefore, it seems that the top five character strengths *fairness, honesty*, *kindness, judgment*, and *love* in the sample of the present study are not that specific for medical professionals but perhaps for the German-speaking population in general. Possibly, socialization in German-speaking countries is particularly oriented toward these character strengths (*fairness* and *kindness* being evident in all samples) as they are perceived as important for human development and cohabitation. When compared to other social professions, in particular physicians’ *honesty* and *kindness* in this sample seemed to be more specific. A recent study investigating character strengths and job satisfaction (Heintz and Ruch, 2020) showed that *fairness, judgment*, and *love* were practically always within the top five character strengths of nurses, teachers, and social workers according to VIA-means, beside *curiosity* and *love of learning*. Only in their sample of nurses, *kindness* was placed second and *honesty* fifth ex aequo with *judgment*. Therefore, this occupational group showed the highest overlap with physicians from this study. Other samples revealed *love of learning* and *social intelligence* to be very important among counselors (compared to a normed sample; Allan et al., 2019) as well as educators, teachers, psychologists, and therapists (Ruch, 2014); *judgment* and *love* were highly evident in teachers and psychologists. Harzer (2008) found in Swiss samples again *fairness* and *love* in teachers and care workers, and *judgment* in psychologists, therapists and social workers. *Curiosity* and *spirituality* were also repeatedly within their top five character strengths. Contrasting the present findings with prior research and deliberations of a cultural basis of character strengths, at least *honesty* can be interpreted as being a more specific strength for medical professionals.

There is evidence, on the one hand, that character strengths overlap with personality traits or occupational interests and on the other that they add incremental validity (e.g., Park et al., 2004; West, 2006). However, individual interests, abilities or skills differed strongly from the conceptual VIA-classification although it is more comprehensive than trait and value taxonomies. Other classifying structures like the Five-Factor model (McCrae and Costa, 1997) or Holland’s RIASEC-model (1997) might help explain underlying patterns of the top five character strengths found in this empirical study. The character strengths *fairness*, *honesty*, *kindness*, and *love* can be assigned to e.g., interpersonal strengths, and *judgment* to intellectual strengths, both significantly correlating with the Big Five dimensions ‘agreeableness’ and ‘openness to experience’ (= being inventive and curious vs. consistent/cautious), while interpersonal strengths were also associated with ‘conscientiousness’ (= being efficient and organized vs. extravagant/careless; Neto et al., 2014). In detail, *fairness* and *kindness* were significantly predicted by ‘agreeableness’ and ‘extraversion’ (= being outgoing and energetic vs. solitary/reserved), with the first strength also being predicted by ‘conscientiousness’ (Noronha and Campos, 2018) and the second by ‘openness to experience’ (Neto et al., 2014). ‘Agreeableness’ was clearly related to *love* (Park et al., 2004) as well as *honesty*, with ‘conscientiousness’ also being relevant for the latter strength (Macdonald et al., 2008), and *judgment* was predicted by ‘openness to experience’ (Neto et al., 2014). Summarizing, ‘agreeableness’ can be considered to be the best predictor of the top five character strengths. However, this dimension is considered to be a superordinate trait, including sub-traits like altruism, compliance, empathy, flexibility, honesty, patience, or trust. The RIASEC types are associated with preferences for vocational activities but also with aversions and by analyzing the description of these traits one can derive ideas on their relationship with certain character strengths. Proyer et al. (2012) described other-directed strengths (*fairness, kindness*), temperance strengths (*honesty*), and transcendence strengths (*love*) coming from the VIA-Youth (Proyer et al., 2012). They showed that ‘social’ interests were predicted by other-directed and transcendence strengths, whereas temperance strengths were correlated with ‘investigative’ interests. Littman-Ovadia et al. (2013) found the same significant correlations for ‘social’ interests but also for ‘artistic’ ones with the respective character strengths, for *judgment* ‘artistic’ and ‘investigative’ interests were relevant but none significantly for *honesty*. According to their results, ‘artistic’ and ‘social’ interests predominate. However, it is important to keep in mind that some character strengths are related to combinations of personality traits or interests and not stand-alone characteristics.

These results might lead to the hypothesis that medical students and resident physicians in this sample tend to be predominantly ‘agreeable’ while having mostly ‘artistic’ and ‘social’ interests. Such people could be described as rather compassionate, cooperative, emotional, friendly, open, and warm. They prefer tasks involving other people and seem to satisfy their needs in helping situations, being in line with literature on the ‘social personality’ including doing good for others (see Littman-Ovadia et al., 2013) and the physicians’ job description.

### Limitations and Implications for Future Research

First, data were self-reported by the participants implying possible bias in terms of distortion effects. In particular, social desirability of certain character strengths might be possible. A former study already showed that especially ‘niceness’ strengths (*fairness, kindness*) but also *honesty* and *love* significantly correlated with social desirability (Macdonald et al., 2008). This is in conflict with Peterson and Seligman (2004) assertion that the VIA-IS is free of social desirability effects because all items are socially desirable. Therefore, additional character strengths assessments by peer-ratings (e.g., friends, family or colleagues) would be helpful. Another issue might be the limited number of participants in some medical specialties. Comparing their respective profiles can only give suggestions or trends and need further evidence. The generalizability of the results is limited due to homogenous sampling (e.g., one culture, same language, similar organizational structures, working climate, etc.). Finally, it might be possible that, in contrast to the assumption of character strengths having a causal impact on well-being, a reverse causality may be present in the data. In a recent study, preliminary evidence is given that (psychological) well-being has a significant positive effect on the applicability of signature character strengths over time indicating that higher levels of well-being might be mandatory first to have access to one’s own signature strengths (Huber et al., 2020). Applicability in this regard refers to i.a. asking whether a character strength is ‘used’ at work or in private life (Harzer and Ruch, 2013). It has to be considered that the level of character strengths and their applicability are different constructs. However, as the VIA-items also contain behavior to some extent, there is a certain overlap enabling possible reverse causal effects.

Future research should focus on a fit between personal characteristics in a more holistic sense. Task-related and social demands in different medical specialties may also warrant future research. Exploring a persons’ narrative, story and other biography processes (see Borges and Savickas, 2002) could further improve the understanding of how medical students and aspiring physicians tick leading to more comprehensive profiles facilitating career decision-making processes, e.g., when knowing that working with other people is a basic interest vs. working with things or data, different sub-disciplines might be recommended. Future research questions could address this issue by e.g., looking at all kinds of demographic factors, and further examining if certain medical specialties can be assigned to more or less people-orientation and if it would be a flaw to be ‘other-oriented’ within the respective discipline. Another research direction should focus on how character strengths could be integrated reasonably in the medical curriculum and further education alongside teaching knowledge and skills. Particularly the hidden medical curriculum, often being contrary to positively valued virtues and different across medical facilities, represents a big issue. When looking through medical oaths there is an extreme variation further undergirding diversity of the hidden curricula (Greiner and Kaldjan, 2018). They need to be uncovered and questioned by educators as well as trainees. Global research on culturally and job-related biased differences in medical professionals’ character strengths profiles would be necessary to positively influence curricula development suitable for respective cultures and adjuvant for (work-related) well-being.

### Conclusion

This study suggests that the character strengths *hope, perseverance, self-regulation, teamwork*, and *zest* are most relevant when it comes to fostering medical students’ and physicians’ well-being and work engagement. However, these were not part of the top five character strengths reported by the medical professionals. Additionally, negative effects of e.g., *bravery, creativity*, or *perspecti*ve on well-being and work engagement were discovered. Creating an institutional environment considering these results could be beneficial for medical professionals’ future well-being and health (Strecker et al., 2019). According to the modern-day physician’s pledge to pay attention to their own health, the recommendation is to promote self-awareness and character building among medical professionals by considering both individual signature character strengths and ‘collective’ profiles. Moreover, research on character strengths profiles in medical professionals’ must also focus on cultural implications with the need for comparing different societies, working cultures, and other parameters of public health systems (e.g., Western vs. Asian culture), focusing on ‘medical common ground’.

## Data Availability Statement

The datasets generated for this study are available on request to the corresponding author.

## Ethics Statement

This study was conducted in accordance with recommendations of ‘The Board for Ethical Questions in Science of the University of Innsbruck’ including written informed consent from all subjects. All participants gave written informed consent subject to the regulations of the Declaration of Helsinki. The protocol was permitted by ‘The Board for Ethical Questions in Science of the University of Innsbruck.’

## Author Contributions

AH, CS, TH, and SH were substantially involved in planning and conducting the study. AH drafted the article. CS and TK carried out the data analyses and reported them. All authors revised the manuscript critically for important intellectual content, read and approved the submitted version.

## Conflict of Interest

The authors declare that the research was conducted in the absence of any commercial or financial relationships that could be construed as a potential conflict of interest.
