# Supplementary material for: Character Strengths Profiles in Medical Professionals and Their Impact on Well-Being
Source: Front Psychol. 2020 Dec 23;11:566728. doi: 10.3389/fpsyg.2020.566728 (PMC7786021; doi:10.3389/fpsyg.2020.566728)
Supplement: Supplementary file 1 [file Table_1.DOCX]

Supplementary Material

# Supplementary Table 1. Comparison of VIA-character strengths between all physicians of different medical specialties

| **VIA-character strengths** | **Anesthesia** | | **Internal Medicine** | | **Psychiatry** | | **Surgery** | |
| --- | --- | --- | --- | --- | --- | --- | --- | --- |
|  | Mean | *SD* | Mean | *SD* | Mean | *SD* | Mean | *SD* |
| Appreciation of Beauty and Excellence | 3.48 | 0.63 | 3.51 | 0.58 | 3.30 | 0.53 | 3.35 | 0.91 |
| Bravery | 3.38 | 0.55 | 3.67 | 0.68 | 3.38 | 0.71 | 3.65 | 0.64 |
| Creativity | 3.38 | 0.52 | 3.55 | 0.58 | 3.28^ps**^ | 0.67 | 3.74^ps**^ | 0.73 |
| Curiosity | 3.86 | 0.52 | 3.82 | 0.53 | 3.74 | 0.52 | 4.00 | 0.47 |
| Fairness | 3.98 | 0.53 | 4.13 | 0.50 | 3.88 | 0.62 | 4.10 | 0.67 |
| Forgiveness | 3.44 | 0.65 | 3.36 | 0.68 | 3.38 | 0.64 | 3.27 | 0.74 |
| Gratitude | 3.53 | 0.61 | 3.37 | 0.65 | 3.32 | 0.56 | 3.51 | 0.77 |
| Honesty | 4.24^ap**^ | 0.39 | 4.32^ip**^ | 0.35 | 3.94^ap** /ip** /ps**^ | 0.46 | 4.37^ps**^ | 0.38 |
| Hope | 3.72 | 0.51 | 3.35 | 0.62 | 3.50 | 0.68 | 3.76 | 0.67 |
| Humility | 3.24 | 0.58 | 3.47^ip*^ | 0.48 | 2.94^ip*^ | 0.64 | 3.25 | 0.62 |
| Humor | 3.78 | 0.66 | 3.52 | 0.77 | 3.35 | 0.56 | 3.70 | 0.72 |
| Judgment | 4.02 | 0.43 | 4.25^ip*^ | 0.47 | 3.84^ip*^ | 0.41 | 4.09 | 0.46 |
| Kindness | 4.18^ap*^ | 0.45 | 4.08 | 0.62 | 3.66^ap*^ | 0.47 | 4.00 | 0.66 |
| Leadership | 3.55^as*^ | 0.49 | 3.78 | 0.60 | 3.47^ps*^ | 0.55 | 3.92^as* /ps*^ | 0.55 |
| Love | 3.90 | 0.62 | 3.88 | 0.69 | 3.90 | 0.63 | 3.95 | 0.66 |
| Love of Learning | 3.68 | 0.67 | 3.72 | 0.62 | 3.87 | 0.48 | 4.02 | 0.70 |
| Perseverance | 3.92 | 0.58 | 3.89 | 0.62 | 3.54^ps*^ | 0.53 | 4.00^ps*^ | 0.50 |
| Perspective | 3.23^as*^ | 0.46 | 3.25 | 0.48 | 3.21 | 0.47 | 3.60^as*^ | 0.61 |
| Prudence | 3.44 | 0.61 | 3.54 | 0.65 | 3.10^ps*^ | 0.39 | 3.62^ps*^ | 0.47 |
| Self-Regulation | 3.02 | 0.71 | 3.17 | 0.70 | 3.03 | 0.56 | 3.09 | 0.69 |
| Social Intelligence | 3.81 | 0.50 | 3.72 | 0.68 | 3.80 | 0.44 | 3.83 | 0.59 |
| Spirituality | 2.33 | 0.87 | 2.38 | 1.19 | 2.36 | 0.91 | 2.23 | 1.04 |
| Teamwork | 3.66 | 0.44 | 3.77 | 0.54 | 3.51 | 0.52 | 3.81 | 0.55 |
| Zest | 3.46 | 0.60 | 3.23 | 0.75 | 3.12^ps*^ | 0.64 | 3.67^ps*^ | 0.64 |

*Note*. SD = standard deviation; ^*^*p* < .05; ^**^*p* < .01; significant mean differences according to ANOVA Bonferroni post hoc test between medical specialties are marked by superscript letters; ^ap^ = anesthesia vs. psychiatry; ^as^ = anesthesia vs. surgery; ^ip^ = internal Medicine vs. psychiatry; ^ps^ = psychiatry vs. surgery; ^ip^ = internal Medicine vs. psychiatry
